# Treatment of Enteric Fever

**Published:** 1892-11-05

**Authors:** 


### The Hospital Clinic.

[*The Editor will be glad to receive offers of co-operation and contributions from members of the profession. All letters should be addressed to* The Editor, The Lodge, Porchester Square, London, W.]

### MIDDLESEX HOSPITAL.

### TREATMENT OF ENTERIC FEVER.

Though it is still regarded as possible that some day a drug may be discovered which may have the power of cutting short or diminishing the severity of enteric fever, at the present time we are far from the knowledge of it, and the treatment of the disease continues to be of the expectant class; at the same time there are many details in connection with it which may bear occasional recapitulation.

As now practised, the treatment of enteric fever at Middlesex Hospital consists in carefully regulating the stimulants and diet so as to maintain the patient's strength, while causing as little disturbance as possible to his digestive tract, and directing attention to the avoidance and remedy of the various complications of the disorder.

The number of cases coming under treatment in the metropolis during the past five years has been much below the average of former periods, and though some of them have been severe, the greater number have been of a mild type, and the mortality less than two-thirds that of the former outbreaks.

Notwithstanding many attempts to vary and extend the diet of typhoid patients, the food considered most appropriate for them consists in the main of milk and beef tea, the latter being discontinued where there is much diarrhœa, the former being always boiled and peptonised, or replaced by whey in cases in which casein appears in the stools.

About two pints of each daily are usually sufficient for an adult; he may be allowed to take more, but should not be suffered to do with less.

More especially in mild attacks, patients sometimes complain of the monotony of this regimen, and some variety may be introduced by allowing jelly, fruit-juice, and even chocolate, finely ground and with the amount of added starch and sugar reduced to a minimum. The majority of patients suffering from enteric fever are so little inclined for food of any sort, and the dryness of the mouth and altered secretions so far destroy the taste, that they accept, without question or interest, such fluids as are provided for them, and there is little temptation under such circumstances to try experiments which can hardly effect any improvement in nutrition, and may easily have very dangerous results.

It is important that the patient should take liquid food at night as well as in the day, and where vomiting or diarrhœa occur, limewater is added with advantage to the milk in the proportion of one to three.

Comparatively few cases pass through an attack without requiring stimulants, and it is the custom to administer them from the commencement instead of waiting for the development of severe symptons before doing so.

Brandy is undoubtedly the best form in which to prescribe alcohol in typhoid, given in divided doses at intervals of two to four hours in half ounce or even smaller doses, the quantity required amounting to two to six ounces daily, though severe cases may require double this amount.

Some caution, however, must be exercised to avoid inducing the narcotic effects of the alcohol, and this can only be done by watching the condition of the circulation and noting the effect of increase or diminution of the dose. Port wine or Burgundy may be given in moderately severe cases, or may be substituted for the spirit towards the end of the attack, but it is well to diminish or omit stimulants as convalescence advances.

Of all diseases, typhoid fever is perhaps that in which good nursing is of the greatest importance—continuous attention and the most absolute cleanliness being essential, not only to the recovery of the individual, but to secure the safety of others. Fortunately instances are not common in which those in attendance contract the disease directly from their charge. Apart from the necessity for the frequent administration of the diluted forms of nourishment and stimulant, and attendance to the urgent wants of the patient, it is absolutely necessary to have a careful record of temperature and of the amount of nourishment taken, and also timely warning of any of the serious symptoms that may arise.

But besides this, there remains much that can be done for the comfort and security of the patient by cleansing and moistening the mouth from time to time with preparations of glycerine, anointing the dry lips with salve to prevent painful fissures and the formation of sores, applying evaporating lotions to the aching head, and in effecting change of posture as a safeguard against bedsores, and to some extent, congestion of the bases of the lungs. It is very much easier to prevent the formation of bedsores by sedulous attention to cleanliness and the avoidance of wet, and by the protection and stimulation of a threatened part, than to arrest extension and promote healing after necrosis has already commenced. In a protracted illness like typhoid fever, a spring or water bed is of great service in maintaining the integrity of the skin where this is threatened, and especially where change of posture is difficult to insure.

Apart from the nursing and general regimen, perhaps that which most frequently calls for special treatment is pyrexia, not only on account of the rapidly wasting effect of fever on the tissues, but because of its peculiar destructive action on the muscles, especially those of the heart. It is not usual to interfere with the course of a temperature which does not exceed 103 in the evening, but in those cases where this persists, or is slightly exceeded, much benefit is derived from "cradling" the patient, that is, arranging a sort of wagon-roof over the bed, covered only with a sheet, and leaving him exposed in the chamber beneath, which may be further cooled by means of ice-bags, until the the temperature is reduced. Rather more severe cases are treated by tepid or cold sponging, the patient being laid on a mackintosh sheet placed over the bed, and rubbed over with a half-filled sponge for ten minutes at a time. This method, which is the one to which least objection is offered, is often very grateful to the patient, producing a cool, moist skin and refreshing sleep.

For a higher temperature, or one that is maintained in spite of these milder measures, one employs either the wet pack, while the patient is wrapped in a saturated sheet over which pieces of ice may be distributed, and which has the advantage of being readily applied; or the cold bath, which, though not widely employed, is certainly the most efficacious of all, and has been the most important agent introduced in recent times in diminishing the mortality in enteric fever, a series of cases in 1884 showing a mortality of 8·3 per cent. in the cases bathed, while it was 17·3 in those not so treated. The cases which are found to give the best results under the bathing treatment are such as commence acutely with severe symptoms in the first week. The bath is long enough to receive the patient at length. He is lifted in by the shoulders and heels, the head resting on an inflated ring air cushion. The temperature of the water is from 70 deg. to 80 deg.

While the patient is in the bath the water is kept agitated by baling till the temperature falls, or is lowered by the addition of ice or cold water 10 deg. during the 10 to 20 minutes immersion; blueness or shivering is regarded as a sign for removing the patient. It is usual to give half an ounce or so of brandy either before, during, or after the bath, to restore the circulation. When the temperature is high late in the disease, and when cases are complicated with hypostatic pneumonia, failing heart, degeneration of muscular tissue, and the more severe abdominal lesions, such as hæmorrhage or perforation, they are obviously not adapted for this treatment. Patients are not generally bathed at a lower temperature than 103·5; not more than 25 baths are required, even in the most severe cases, and three is the greatest number administered in one day. Antifebrin and antipyrin in 5 and 15 grain doses respectively, produce in some cases temporary effect in reducing fever, but their benefit in this direction seems more than counter-balanced by the irritation they cause in the digestive tract and their depressing action on the heart. The severe abdominal complications, hæmorrhage, and perforation are best treated exclusively by opium.

Provided the tongue is clean when the fever declines and convalescence commences, custard pudding and rusks are added to the dietary on the following day, and if all goes well, boiled fish within a week and meat a week later.

The patient has been so long without solid food that he will not bear much delay, but as the commencement of feeding is sometimes followed by relapse, even more severe than the original attack, great caution is always exercised in arranging it, and any rise of temperature is accepted as a warning.

